# Lessons learned from the COVID-19 pandemic: identifying hesitant groups and exploring reasons for vaccination hesitancy, from adolescence to late adulthood

**DOI:** 10.3389/fpubh.2024.1456265

**Published:** 2024-12-24

**Authors:** Laure Pauly, Caroline Residori, Hamid Bulut, Dmitry Bulaev, Soumyabrata Ghosh, Marc P. O’Sullivan, Joëlle V. Fritz, Michel Vaillant, Basile Rommes, Robin Samuel, Venkata P. Satagopam, Rejko Krüger, Anja K. Leist, on behalf of the CON-VINCE Consortium, the ORCHESTRA Working Group

**Affiliations:** ^1^Transversal Translational Medicine, Luxembourg Institute of Health, Strassen, Luxembourg; ^2^Faculty of Science, Technology and Medicine, University of Luxembourg, Esch-sur-Alzette, Luxembourg; ^3^Luxembourg Centre for Systems Biomedicine, University of Luxembourg, Esch-sur-Alzette, Luxembourg; ^4^Parkinson Research Clinic, Centre Hospitalier de Luxembourg, Strassen, Luxembourg; ^5^Department of Social Sciences, University of Luxembourg, Esch-sur-Alzette, Luxembourg; ^6^Competence Centre for Methodology and Statistics, Luxembourg Institute of Health, Strassen, Luxembourg

**Keywords:** vaccine education, health promotion, public health, vaccination hesitancy, vaccination willingness, COVID-19, immunity

## Abstract

**Introduction:**

The COVID-19 (COronaVIrus Disease-2019) pandemic highlighted the importance of assessing the rationales behind vaccine hesitancy for the containment of pandemics. In this nationwide study, representative of the Luxembourgish population, we identified hesitant groups from adolescence to late adulthood and explored motivations both for and against vaccination.

**Methods:**

We combined data collected via online surveys for the CON-VINCE (COvid-19 National survey for assessing VIral spread by Non-affected CarriErs) study, 1865 respondents aged 18–84, and for the YAC (Young people And Covid-19) study, 3740 respondents aged 12–29. Data from both studies were harmonized and weighted to ensure a sample representative of Luxembourg’s resident population. The surveys included information on demographic and socio-economic factors as well as vaccination hesitancy.

**Results:**

At the time of the survey, 67.0% of respondents had been vaccinated against SARS-CoV-2 (Severe Acute Respiratory Syndrome COronaVirus-2), while 33.0% of the respondents had not yet been vaccinated. Of those not yet vaccinated, 41.8% of respondents were vaccine hesitant. The most important concerns against vaccination were that the vaccine had not been tested sufficiently (59.4%) and the fear of side effects (52.4%). The most frequent reasons for vaccination were to help society overcome the pandemic (74.8%), and to protect oneself from the consequences of infection with the virus (69.3%). The proportion of unvaccinated respondents unwilling or undecided to get vaccinated was higher in the younger age groups compared to the higher age groups.

**Conclusion:**

Our findings contribute to improving public health policy communications, not only for future pandemics but also for routine vaccination campaigns. This will help reach those who are unwilling (26.7%) or undecided (15.1%) about vaccination and reinforce strategies that have successfully increased vaccination willingness.

## 1 Introduction

In the context of the COVID-19 (COronaVIrus Disease-2019) pandemic, vaccination played a crucial role in enabling society to return to a normal pattern of life and in maintaining this normalcy during future seasonal outbreaks. After the declaration of the SARS-CoV-2 (Severe Acute Respiratory Syndrome CoronaVirus-2) virus outbreak as a pandemic by the World Health Organization (WHO) in March 2020 (1), COVID-19 vaccines were developed rapidly and proved to be highly effective. Studies have demonstrated that vaccine effectiveness varied across different waves of the pandemic (2). A recent WHO/Europe study revealed that COVID-19 vaccinations were associated with a significant reduction in mortality, saving over 1.6 million lives. Most of these lives were saved during the period when the Omicron variant was dominant, from December 2021 to March 2023 (3). In Luxembourg, the context of our study, roughly half a year after the first administration of a COVID-19 vaccine at the end of December 2020 (4), all adult residents had received an invitation for a free vaccination against SARS-CoV-2 by July 07, 2021 (5). Invitations for 12- to 17-year-old residents were sent from June 28, 2021 onwards (6) (Supplementary Table A.2). Without a legal framework for compulsory vaccination, the success of vaccination campaigns depends on individual willingness to be vaccinated. Previous surveys have shown high COVID-19 vaccination willingness in Luxembourg, with 82 to 86% of the adult population willing to be vaccinated against SARS-CoV-2 in mid-2021. The numbers were similar in Germany (85%) but slightly lower in France (79%) (7–9). Despite this high reported vaccination willingness and the easy access to vaccinations via numerous vaccination centers, data (October 05, 2023) from the European Centre for Disease Prevention and Control (ECDC) statistics show that only 72.7% of the total Luxembourgish population are fully vaccinated against SARS-CoV-2 (primary course). This vaccination rate is slightly below European Union (EU) / European Economic Area (EEA) average of 73.0%. With a higher proportion of the Luxembourgish population (57.9%) obtaining an additional first “booster” dose, compared to 54.8% in the EU/EEA (10).

Despite vaccination being recognized as one of the most successful public health interventions, some groups of individuals are skeptical and choose to delay or refuse vaccines. The Strategic Advisory Group of Experts on Immunization (SAGE) Working Group on Vaccine Hesitancy defined vaccine hesitancy as delay in acceptance or refusal of vaccination despite availability of vaccination services (11). In Luxembourg, surveys done in mid-2021 revealed that 13 to 14.5% of respondents did not intend to get vaccinated against SARS-CoV-2. Numbers on vaccine reluctance were similar in Germany (13%) and considerably higher in France (21%) (7–9).

To increase vaccination rates in the context of an epidemic or pandemic, public health messaging needs to target those who are undecided about vaccination, as this population may be convinced to be vaccinated compared to those unwilling to get vaccinated. Against this backdrop, we aimed to explore motivations for and against vaccination, from adolescence to late adulthood drawing on a representative sample of residents across Luxembourg. Our results can help adapt public health policy communication in future pandemics, to reach those unwilling or hesitant to be vaccinated, and to reinforce those strategies that may increase vaccination confidence.

## 2 Materials and methods

### 2.1 Study design and participants

This study analyzes data collected from two studies using harmonized survey questions.

The recruitment of young participants from 12 to 29 years of age took place within the framework of the *YAC* (Young people And Covid-19) study, a nationally representative study about young people and COVID-19, aiming to identify the social, economic and health consequences of the pandemic in the younger population. A sample of the resident population stratified by gender, age and canton of residence, was drawn using simple random sampling from the National Registry of Natural Persons (RNPP) for the YAC 2021 cross-sectional survey (12). A total of 3,740 respondents participated from August to September 2021.

The recruitment of participants aged 18 to 84 years, took place in the framework of the nationally representative observational *CON-VINCE* (COvid-19 National survey for assessing VIral spread by Non-affected CarriErs) study, aiming to evaluate the prevalence of SARS-CoV-2 infections in the adult population. The sampling strategy aiming for representativeness of the Luxembourgish population was based on stratification by gender, age, and canton of residence and was realized in collaboration with a specialized survey company (TNS-ILRES) within their respondent panel. The full CON-VINCE study protocol and cohort have been reported in detail elsewhere (13). Participants of the CON-VINCE study were followed up for over 12 months. In the current study, we analyze and discuss the latest follow-up assessment between April and June 2021 with a total of 1,578 respondents.

### 2.2 Data protection procedure

In the *YAC* study, in collaboration with the Centre des Technologies de l’Information de l’État (CTIE), individuals from a proportionally stratified random sample, registered at the RNPP were selected and received a sequential identification number. Selected individuals were contacted by the CTIE via personalized postal invitations. In the invitation letters, selected respondents were informed about the aims of the project and the data protection guidelines. Data was collected using a secure web interface hosted by QualtricsXM and stored on secure servers at the University of Luxembourg.

In the *CON-VINCE* study, participants were recruited in collaboration with the survey company based on a large representative panel of residents of Luxembourg. To allow data collection in a pseudonymized manner, a persistent identifier was assigned to each participant. Data were collected via a secure web interface and were stored on a secure data platform at the Luxembourg Centre for Systems Biomedicine (University of Luxembourg).

### 2.3 Ethics

The *YAC* study was approved, as required for social sciences surveys conducted by the University of Luxembourg, by the Ethics Review Panel. Ethics approval was obtained for the study on 18 June 2021 (20-041-C-A (YAC+ (amendment 1))). In accordance with current regulations, the Commission Nationale pour la Protection des Données was notified prior to conducting the study. On the survey platform, all selected respondents gave consent before filling out the questionnaire (i.e., they had to explicitly agree to the privacy terms and conditions of the survey). The survey’s data protection policy was provided in French, Luxembourgish and German. For respondents below the age of 16 the consent of the respondents’ legal guardians was ensured by sending the invitation letters to them. As an incentive, vouchers worth €10 were offered to the first 2,000 participants upon completion of the questionnaire. Respondents were not requested to provide personal details for receiving the vouchers at any time (12).

The *CON-VINCE* study was conducted according to the Ethical Principles for Medical Research Involving Human Subjects, as stated in the 2013 revised version of the 1964 World Medical Association Declaration of Helsinki and registered under clinicaltrials.gov under NCT04379297. The national research ethics committee (Comité National d’Ethique de Recherche, CNER) and the Luxembourgish Ministry of Health (references 202004/01 and 831x6ce0d, respectively) approved the study. All participants completed an electronic informed consent form and had the right to withdraw from the study at any time. Further details are described in Tsurkalenko et al. (13).

### 2.4 Measurements

The surveys of both studies included questions about demographic and socio-economic information and information about vaccination willingness/hesitancy and their vaccination status (at least partially vaccinated). Both studies used the same questions regarding vaccination.

The reasons for vaccination willingness were asked using the question: “What are the reasons why you will agree to get vaccinated against coronavirus/COVID-19?,” followed by 8 reasons that could be selected if they apply (Table 1).

**Table 1:** Questions and answers regarding vaccination willingness and hesitancy for CON-VINCE and YAC.

CON-VINCE study	YAC study
Vaccination willingness	
Will you agree to get vaccinated against COVID-19 when it is your turn?	Will you get vaccinated against coronavirus/COVID-19?
What are the reasons for you to agree to get vaccinated against COVID-19?	
I want to protect myself	I want to protect myself
I want to protect a vulnerable significant other	I want to protect my vulnerable partner
	I want to protect somebody who I am close to
I want to help our society combat the pandemic	I want to help our society combat the pandemic
It is recommended by the government	It is recommended by the government
My treating physician told me to do so	My treating physician told me to do so
It is recommended by my employer	It is recommended by my employer
I think vaccination is important in order to be able to travel safer	I think vaccination is important in order to be able to travel safer
Other reasons	I hope that a vaccination will offer greater freedom
	Other reason (free text*)
Why are you undecided about getting vaccinated against COVID-19?	
I do not believe in vaccinations in general	I do not think much of vaccinations in general
I also do not get vaccinated against other diseases	I also do not get vaccinated against other diseases
I have had bad experiences with other vaccinations	I have had bad experiences with other vaccinations
I do not feel well enough informed about vaccinations in general	I do not feel well enough informed about vaccinations in general
I do not feel well enough informed about COVID-19 vaccinations	I do not feel well enough informed about COVID-19 vaccinations
I do not think I need a vaccination against COVID-19 because I am not in the risk group	I do not think I need a vaccination against COVID-19 because I am not in the high-risk group
I prefer to wait until more people have been vaccinated	I prefer to wait until more people have been vaccinated
I am afraid of possible side effects	I am afraid of possible side effects
I am sceptical that the COVID-19 vaccine really protects	I am sceptical that the COVID-19 vaccine really protects
I am afraid that COVID-19 vaccine does not protect against future mutated forms of the Coronavirus	I am afraid that COVID-19 vaccine does not protect against future mutated forms of the coronavirus
I think that the vaccine has not been tested sufficiently	I think that the vaccine has not been tested sufficiently
Other reasons	Other reason (free text*)
	The vaccination is not currently approved for my age group
Why do you think it is unlikely for you to agree to get vaccinated against COVID-19?	
I do not believe in vaccinations in general	I do not think much of vaccinations in general
I also do not get vaccinated against other diseases	I also do not get vaccinated against other diseases
I have had bad experiences with other vaccinations	I have had bad experiences with other vaccinations
I do not feel well enough informed about vaccinations in general	I do not feel well enough informed about vaccinations in general
I do not feel well enough informed about COVID-19 vaccinations	I do not feel well enough informed about COVID-19 vaccinations
I do not think I need a vaccination against COVID-19 because I am not in the risk group	I do not think I need a vaccination against COVID-19 because I am not in the high-risk group
I prefer to wait until more people have been vaccinated	I prefer to wait until more people have been vaccinated
I am afraid of possible side effects	I am afraid of possible side effects
I am sceptical that the COVID-19 vaccine really protects	I am sceptical that the COVID-19 vaccine really protects
I am afraid that COVID-19 vaccine does not protect against future mutated forms of the Coronavirus	I am afraid that COVID-19 vaccine does not protect against future mutated forms of the coronavirus
I think that the vaccine has not been tested sufficiently	I think that the vaccine has not been tested sufficiently
Other reasons	Other reason (free text*)
	The vaccination is not currently approved for my age group

*Respondents had the option to explain their other reasons in their own words in the YAC study. These explanations were recoded to fit the answer options and/or counted as “other reasons”.

The reasons for vaccination hesitancy were asked using the question: “Why [are you undecided about getting/do you think it is unlikely for you to agree to get] vaccinated against coronavirus/COVID-19?,” followed by 12 reasons that could be selected if they apply (Table 1).

Given the multilingual nature of Luxembourg, the questionnaire in CON-VINCE was administered in German, French, English, and Portuguese in both studies and additionally in Luxemburgish in the YAC study.

### 2.5 Statistical analyses

Data from both studies were merged and weighted to represent the resident population of Luxembourg (as of January 01, 2021) as estimated by the Institut National de la Statistique et des Etudes Economiques du Grand-Duché de Luxembourg (14). The method of weighting used was post-stratification with finite population correction, using the complete age-by-gender-by-canton population distribution.

A table of baseline demographic sample characteristics was provided, where categorical variables were described as frequencies (n), and sample proportions (%), while the continuous variables were summarized as median (IQR). The categorical factors were compared between the two cohorts through the Fisher’s exact tests. For the continuous variables, Wilcoxon-Mann–Whitney tests were performed. All tests were two-tailed and a *p*-value below 0.05 was considered statistically significant.

**Figure 1:**
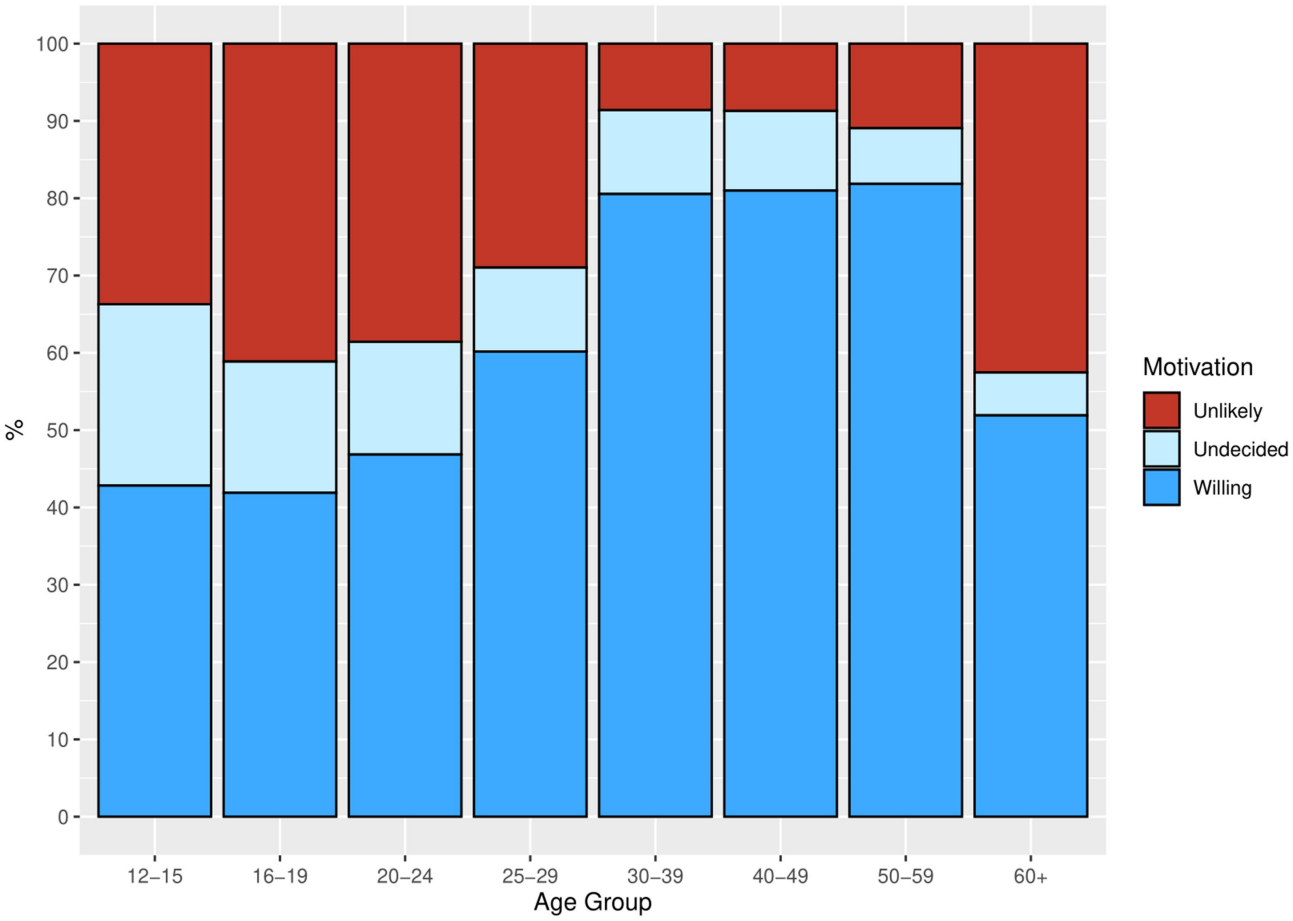
Bar chart representing vaccination willingness and hesitancy in unvaccinated by age group. Source: YAC 2021 (*n* = 814); CON-VINCE April–June 2021 (*n* = 760) weighted dataset.

Vaccination intention by age group was summarized in a bar chart using the population-weighted dataset as shown in Figure 1.

**Figure 2:**
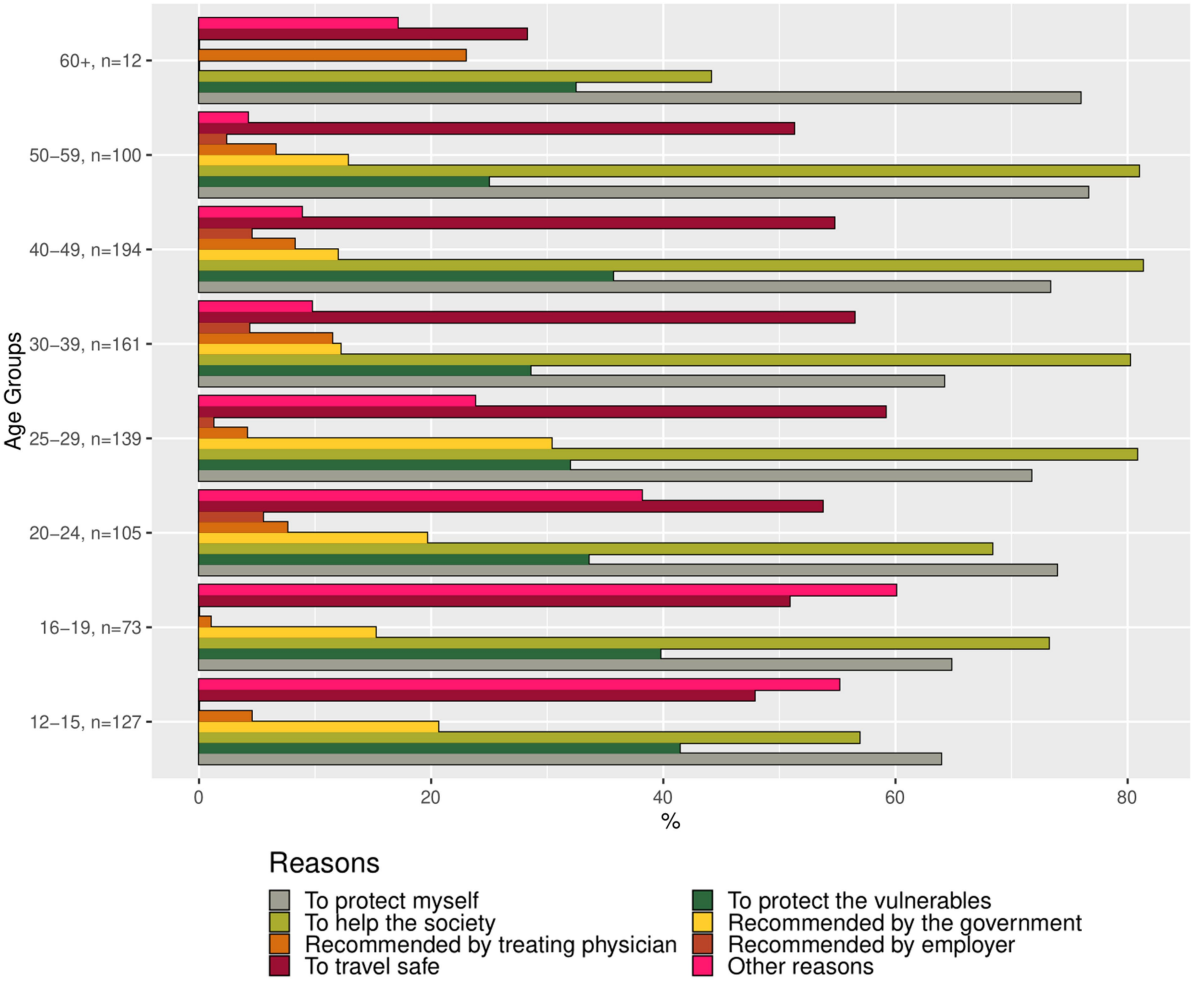
Bar chart representing the reasons for vaccination willingness among unvaccinated individuals, categorized by age group (proportions of respondents). Source: YAC 2021 (*n* = 298); CON-VINCE April–June 2021 (*n* = 613). Weighted summaries, multiple responses were possible.

**Figure 3:**
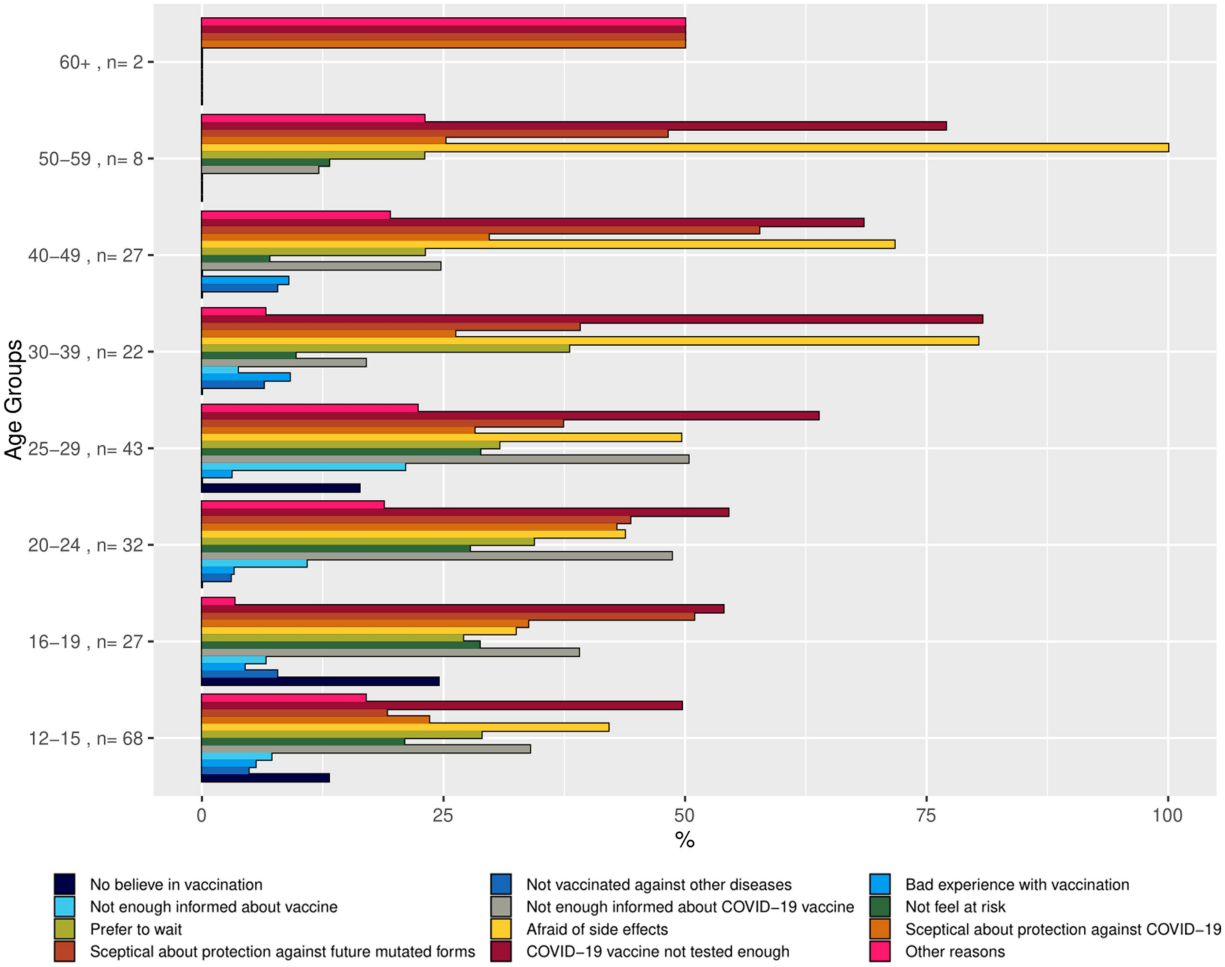
Bar chart representing reasons for vaccination hesitancy (undecided) by age group (proportions of respondents in the age group indicating the reason). Source: YAC 2021 (*n* = 164); CON-VINCE April–June 2021 (*n* = 65). Weighted summaries, multiple responses were possible.

**Figure 4:**
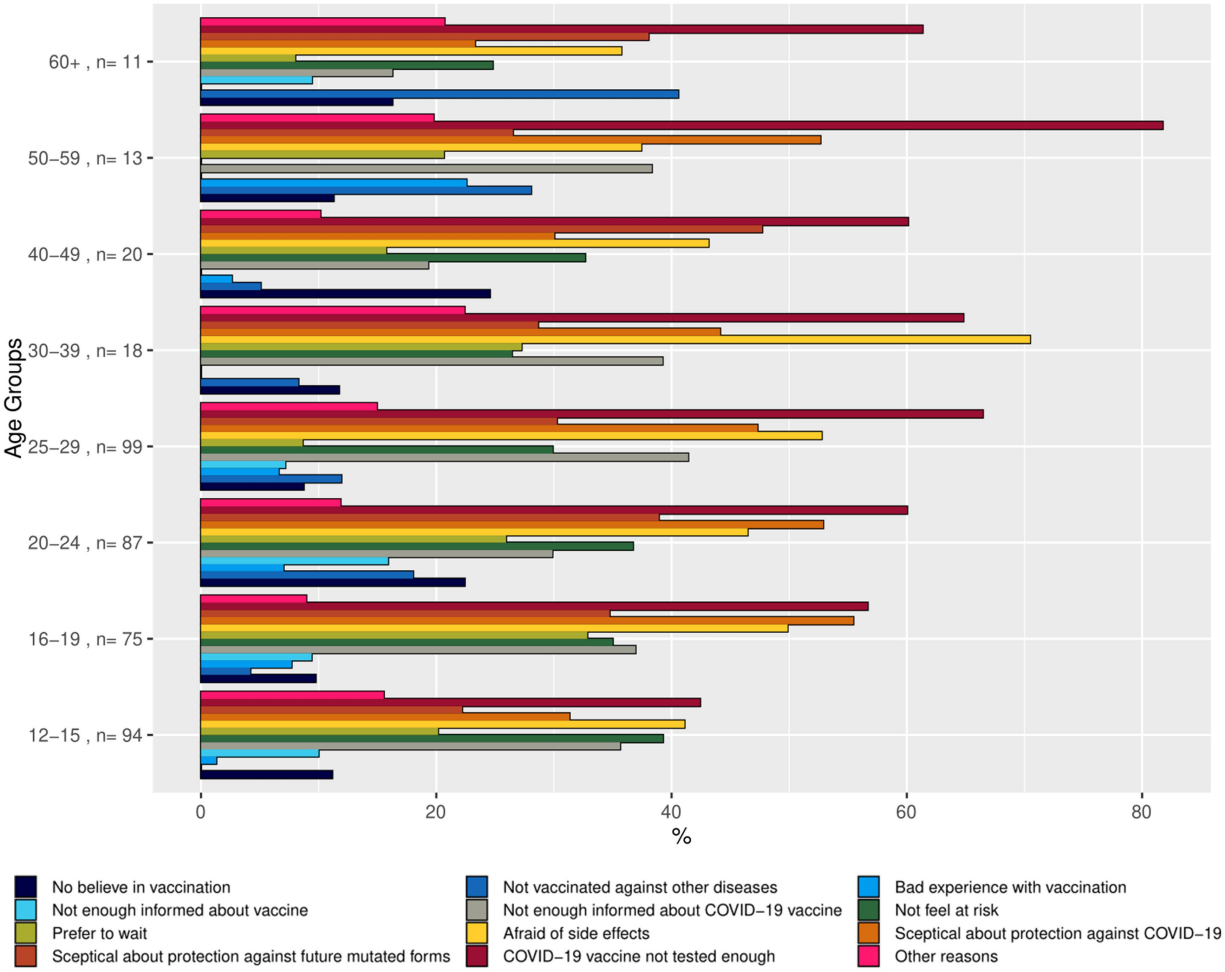
Bar chart representing reasons for vaccination unlikeliness by age group (proportions of respondents in the age group indicating the reason). Source: YAC 2021 (*n* = 340); CON-VINCE April–June 2021 (*n* = 77). Weighted summaries, multiple responses were possible.

We hypothesized that there are specific motivations that explain why individuals are hesitant to get vaccinated. The reasons for vaccination willingness or vaccination hesitancy were presented as bar charts, using the weighted summaries (Figures 2–4). The corresponding tables on the reasons for vaccination willingness/hesitance, with crude sample frequencies and sample proportions, as well as with the population prevalence estimated through weighting are provided in Supplementary Tables A.4.1-A.4.3.

**Figure 5:**
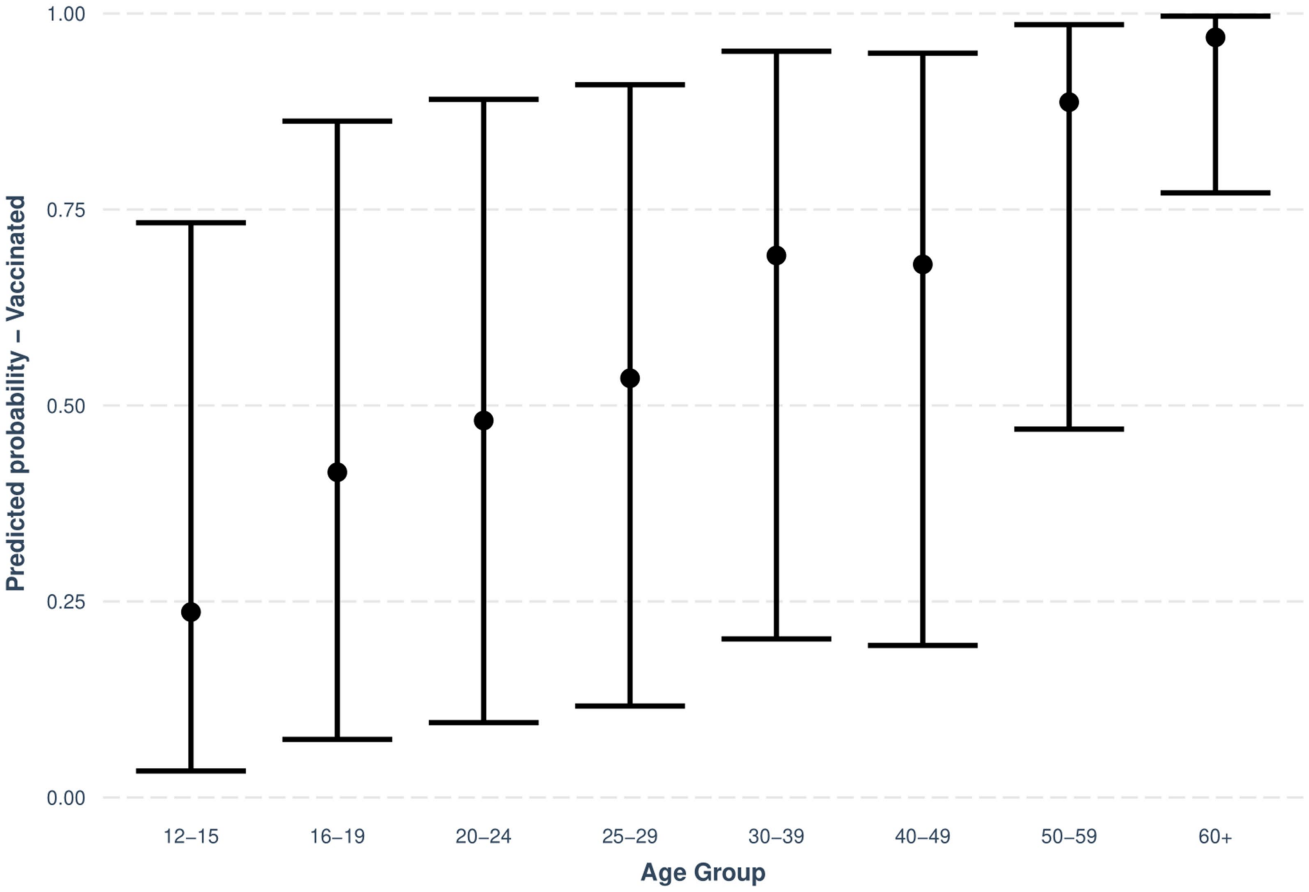
Predicted probabilities with 95% CIs for vaccination status by age group. Source: YAC 2021 (*n* = 3160); CON-VINCE April–June 2021 (*n* = 1,576).

Vaccination status within each level of the categorical variables (age group, gender, employment status, migration background) were summarized as frequencies (*n*) and sample proportions (%) and are provided in Supplementary Table A.1. A generalized linear mixed effects model (GLMM) was built to further analyze the relationship between the vaccination status and sociodemographic factors. We hypothesized that specific socio-economic factors might be associated with vaccination status. The outcome was a binary variable describing the vaccination status; the fixed effects variables were categorical sociodemographic factors (age group, gender, employment status, migration background), while the study effect (CON-VINCE/YAC) was considered as a random effect. The reported odds ratios from the GLMM are Conditional Effects (Supplementary Table A.1). The predicted probabilities of vaccination status by age group were obtained from the model. These probabilities are presented in a plot with 95% confidence intervals (Figure 5). Data analyses were performed using R (version 4.2.1).

## 3 Results

In a first step, we described the demographic characteristics of both cohorts (Supplementary Table A.0). In a second step, we described the vaccination status and its associations with sociodemographic factors. We visualized the predicted probabilities of vaccination status by age group after adjusting for gender, employment status, migration background and the random effect of study (CON-VINCE/YAC) in the model (Figure 5; Supplementary Table A.1). In a third step, we described vaccination intention for the different age groups (Figure 1) and reasons for vaccination willingness (Figure 2) and hesitancy (Figures 3, 4).

### 3.1 Vaccination status and associations with sociodemographic factors

Of the 4,760 respondents with data on vaccination, 3191 (67.0%) respondents had already received at least one COVID-19 vaccination, whilst 1,569 (33.0%) respondents had not been vaccinated at the time of the survey.

Figure 5 shows the predicted probabilities of vaccination status by age group when adjusting for gender, employment status, migration background and study random effect in the model. The older the age group, the higher the predicted proportion of vaccinated respondents in the age group.

Results from the regression model that includes age group, gender, employment status, and migration background (Supplementary Table A.1) show that sociodemographic factors such as age group, employment status, and migration background are statistically significantly associated with vaccination status (*p* < 0.05), while gender is not related to vaccination status (*p* ≥ 0.05).

The association between age group and vaccination status in this model was statistically significant at a *p*-value of <0.001 for every age group with higher age being associated with higher probability of being vaccinated. The odds ratio of being vaccinated in comparison to the 12- to 15-year-old reference group range increased with age from 2.29 (95% CI: 1.82–2.88) for the 16- to 19-year-olds to 102.94 (95% CI: 48.38, 219.01) for the respondents in the 60+ age group.

For employment status, being unemployed, or being in retirement were statistically significantly associated with the vaccination status in this model. Respondents who were unemployed (OR: 0.49, 95% CI: 0.34–0.71) had a lower probability of being vaccinated than the full-time employed reference group. Respondents who were in retirement (OR: 3.26, 95% CI: 1.80, 5.93) had a higher probability of being vaccinated than the reference group.

Migration background was statistically significantly associated with vaccination status in this model for all respondents. Compared to respondents reporting Luxembourg as their country of origin (i.e., native participants), respondents reporting their country of origin to be a Southern European country (IT, PORT, ESP) (*p*-value <0.001), a country belonging to the category “other” (*p*-value <0.001) or a Western European country (DE, NL, FR, BE) (*p*-value <0.01) had a lower probability of being vaccinated.

### 3.2 Vaccination intention and reasons for vaccination intention

Of the 1569 respondents who were not yet vaccinated at the time of the survey, 913 (58.2%) indicated that it is very or rather likely that they will get vaccinated, 419 (26.7%) indicated that it is very or rather unlikely that they will get vaccinated, and 237 (15.1%) respondents indicated that they did not yet know whether they will get vaccinated (Supplementary Table A.3).

In the weighted sample, representing the actual population, this corresponds to 73.7% [95% CI: 70.8–76.5] of individuals willing to vaccinate, 15.3% [95% CI: 13.2–17.6] unlikely to vaccinate, and 11.0% [95% CI: 9.1–13.1] of those who are undecided about vaccination (Supplementary Table A.3).

The proportion of unvaccinated respondents not willing or undecided to get vaccinated in the future is higher in the younger age groups than in the older age groups (Figure 1). These results do not seem to apply to the 60+ age group, but most of the participants belonging to this age group were already vaccinated (see Figure 1). The sample of participants in 60+ age group that has not been vaccinated is quite small, n = 25. Therefore, reasons for vaccine willingness/hesitancy in this subgroup should be interpreted with caution.

#### 3.2.1 Reasons for vaccination willingness

Nine hundred and thirteen respondents were not yet vaccinated at the time of the survey but indicated that it was likely that they would get vaccinated in the future. Figure 2 shows the proportions of respondents who indicated a certain reason for their vaccination willingness by age group. Of those 913 participants willing to get vaccinated, 911 provided reasons for their willingness. The weighted and unweighted summaries on the corresponding reasons are given in Supplementary Table A.4.1.

Out of 911 respondents, a total of 681 (74.8%) reported the altruistic motivation “to help the society overcome the pandemic,” corresponding to 78.9% [95% CI: 75.2–82.1%] in the weighted analyses. This was the most named reason in those respondents who were willing to get vaccinated. For respondents from the age groups 12–15 and 20–24 as well as 60+ the self-preserving motivation “to protect myself” was the most named reason to get vaccinated. In total 631 respondents (69.3%) reported this reason, with the estimate for the population of 70.5% [95% CI: 66.5–74.2%]. Among the younger age groups, 12–29, a higher proportion of respondents indicated “recommended by the government,” ranging from 15.2 to 30.4% in the weighted sample, and “other” reasons, in the range of 23.8–60.1% in the weighted sample, as reasons for their vaccination willingness than in the older age groups. The prevalence of these reasons in the weighted sample in respondents of age 30+ was 0.0–12.8% for “recommended by the government” reason, and 9.7–17.1% for “other” reasons (Figure 2).

#### 3.2.2 Reasons for vaccination hesitancy: undecided to get vaccinated

A total of 237 respondents were not yet vaccinated at the time of the survey and indicated that they did not know whether they would be vaccinated in the future. Figure 3 shows the proportions of respondents who indicated a certain reason for their vaccination undecidedness by age group, and Supplementary Table A.4.2 contains the statistics on the reasons in the weighted and unweighted sample.

Of those 237 participants undecided to receive a vaccine, 229 provided reasons for their undecidedness. Out of 229 participants, a total of 120 participants (52.4%) reported to be afraid of side effects, or, 67.9% [95% CI: 58.9–75.7%] in the weighted sample. The proportion indicating being afraid of side effects in the weighted sample was higher among the older age groups than the younger age groups; by 71.6—100.0% in those of age 30–59 against 32.7–49.6% in those of age 12–29 (Figure 3). One hundred and thirty-six participants (59.4%) reported that they fear that the COVID-19 vaccine had not been tested enough, with the population estimate of 68.9% [95% CI: 59.9–76.7%]. This reason for being undecided was especially often indicated by the middle age groups aged from 30 to 59, varying from 77.0 to 80.8% after weighting (Figure 3). Reasons referencing a perceived lack of information on vaccinations in general and COVID-19 vaccinations were indicated more frequently by respondents in the younger age groups. Similarly, the younger age groups seem to feel less “at risk” than the older age groups. Namely, the weighted prevalence of respondents in the age groups 12–29 declaring they do not feel “at risk” was varying between 20.9 and 28.8%, while in the groups of 30 years or older the range was between 9.7 and 13.2% (Figure 3).

#### 3.2.3 Reasons for vaccination hesitancy: unlikely to get vaccinated

419 respondents were not yet vaccinated at the time of the survey and indicated that it was unlikely that they would receive the vaccine in the future. Figure 4 shows the proportions of respondents who indicated certain reasons for their vaccination reluctance by age group, and Supplementary Table A.4.3 contains the statistics on the reasons in the weighted and unweighted sample.

Of those 419 respondents unwilling to get vaccinated, 413 provided reasons for their unwillingness to get vaccinated in the future. Of those, a total of 237 (56.8%) reported that COVID-19 vaccines have not been tested enough as reason for their reluctance, 190 (45.6%) reported fear of side effects, and 190 (45.6%) reported being skeptical about the effectiveness in terms of COVID-19 protection as reason. The corresponding population estimates on the prevalence of those three reasons were 62.1% [95% CI: 54.7–69.0%], 48.6% [95% CI: 41.1–56.2%], and 41.9% [95% CI: 34.7–49.5%] respectively.

## 4 Discussion

In this study, we explored motivations for and against COVID-19 vaccination in the Luxembourgish population and compared vaccination hesitancy and its associated factors between adolescence and late adulthood. Understanding the critical reasons influencing the acceptance of the COVID-19 vaccines will help inform public health policy communications for future pandemics but also for regular vaccination campaigns.

In our age-comprehensive sample ranging from adolescence to late adulthood, 67.0% of the respondents were already vaccinated at time of data collection. Of the respondents that were not yet vaccinated at the time of the survey, 58.2% were willing to receive the COVID-19 vaccine, 15.1% were still undecided and 26.7% did not intend to get a COVID-19 vaccination. When drawing references from the weighted analyses about the actual population, there were 73.7% willing to get vaccinated, 11.0% undecided about vaccination, and 15.3% unlikely to be vaccinated individuals.

According to the SAGE Vaccine Hesitancy Working Group and the European Centre for Disease Prevention and Control (ECDC) reasons against vaccinations are diverse and depend on time and regions, with fear of side effects, perceived low risk of vaccine preventable disease, and mistrust in health care providers to be the most common sources for vaccine hesitancy. The SAGE Working Group concluded that vaccine hesitancy is influenced by factors such as complacency [do not perceive a need for a vaccine, do not value the vaccine], convenience [access] and confidence [do not trust vaccine or provider] (11). We confirmed some of these insights in the Luxembourgish sample ranging from adolescence to late adulthood: (A) The most prevalent reasons against vaccination were the fear of long-term-side effects and the fear that the vaccine has not been tested sufficiently. (B) The most prevalent reasons for vaccination were an altruistic motivation, specifically to help society overcome the pandemic, and the self-serving motivation to protect oneself from the virus.

Vaccine hesitancy in Europe is not a recent occurrence. It has a long history, dating back to the early 19^th^ century when the smallpox vaccine was introduced (15). During this period, there was already a resistance to vaccination, fuelled by fears and misconceptions about the new medical intervention. Over the years, vaccine hesitancy has persisted and evolved, up to recent times, when the WHO has recognized vaccine hesitancy as one of the most serious threats to global health (16). Between 2018 and 2020, public perception of vaccines across the EU improved significantly, particularly for the seasonal influenza vaccine. However, from 2020 to 2022, these positive perceptions have reversed, with declines in the perceived importance, safety, and effectiveness of vaccines (17). Despite well-documented evidence of the effectiveness and safety of vaccines, including COVID-19 vaccines, many people continue to doubt their effectiveness and safety. Enhancing public awareness about the nature of vaccine side effects may have an important impact on vaccine hesitancy (18) and can help reduce misinformation. By providing clear and accurate information about what possible side effects to expect and how common they are, health authorities can build greater trust in vaccines (19). Public education campaigns explaining the rigorous processes involved in vaccine development and approval can also reassure the public about vaccine safety.

Older age groups have consistently shown higher vaccine confidence compared to younger age groups, however this gap in confidence seems to be widening after the pandemic across most EU states (17). Additionally, our study indicates that vaccine hesitancy is more prevalent among the younger population. This could be explained by the fact that younger respondents perceive themselves as less susceptible to COVID-19 than the individuals in late adulthood. Even though the younger population experience mostly mild symptoms of the disease, they can develop long COVID or post COVID condition (20). Furthermore, with 15–42% of the younger population being asymptomatic, the younger population can unknowingly spread the virus (21). Furthermore, reasons referencing a perceived lack of information on vaccinations in general and COVID-19 vaccinations are indicated more frequently in the younger generation than in individuals in later adulthood. The information distributed by the government and the media might have reached the younger generation less effectively than the older generation and there may be a need to expand traditional health communication to include tools that reach the younger generation and messages that are attractive for and tailored to them (22). An additional challenge for vaccination in this group is the fact that children are legally dependent on their parents or guardians. Therefore, the opinion of parents towards vaccination is an important factor in getting COVID-19 vaccinations to the younger age groups. We hypothesize that lower prevalence of vaccine hesitancy in individuals in late adulthood could also be explained by the fact that individuals in late adulthood have already experienced positive impacts of the vaccination and better understand its importance (e.g.: polio vaccine) than the younger generations. Overall, we concur with the statement that further research is needed to determine whether the observed declines in vaccine confidence among the younger population are temporary or indicative of a more enduring public health challenge (23).

To increase vaccination rates in a high vaccination-readiness country, public health messaging needs to target those unsure or rather unwilling to be vaccinated, as they are most likely to be convinced to be vaccinated. Especially the younger generation should be targeted. Concerns especially about vaccine safety and efficiency underline the need for providing clear, understandable information about the principles of vaccines, its risks, and its benefits for the individual as well as also for the population. Furthermore, given that self-protection is one of the most prevalent reasons for vaccination, public health should highlight in their vaccine communication strategies the effectiveness of the vaccine especially in the context of long-COVID. Our findings also allude to optimal communication channels for public health messages. Other studies found that most people use social media as their main source for COVID-19 information and that it is an effective dissemination route for key information (24). Given that infodemics are an important problem in the digital era, propagation of false claims and misinformation that erode trust in institutions and health policies, must be avoided (25). These channels should, in contrast, be used to spread validated information on vaccination and guidelines on the SARS-CoV-2 virus, especially to reach younger generations who, based on our findings, do not feel sufficiently informed (22). Furthermore, the importance of digital verification services has grown over time. Global fact-checking organisation, with specialised journalists, exist to monitor online content and to verify false claims, misinformation, and refute them by retrieving the original or explaining the true facts (26). These fact checking activities should be better promoted to enhance the impact of public health messages. Research should investigate how vaccine-specific misinformation affects vaccine confidence and develop strategies to strengthen resilience to misinformation among the public (23).

Previous findings reported that during the first lockdown, in comparison to other European countries, Luxembourg residents had the highest confidence in their government and health services to deal with the health crisis (27). This highlights the importance to maintain trust in the government and health system. Previous studies have supported the idea that information related to COVID-19 should be delivered by a trusted expert, e.g., general practitioners (28), especially as findings suggest that recommendation from health professionals are positively associated with vaccine uptake. Nevertheless, in our study, only a few respondents indicated recommendation by a practitioner as a reason for vaccination willingness.

Reasons for comparatively high vaccination willingness could be related to the organizational and educational measures by the Luxembourgish public health system. With a population of approximately 650.000 over 2,586 km^2^, Luxembourg’s small size facilitated crisis management by easing the implementation of measures. However, its openness, cultural diversity, and reliance on foreign workers posed challenges for educational continuity and key sectors. The stable political system and centralized governance enabled quick government decision-making (29). Specifically, the Luxembourgish government established a vaccination campaign to increase vaccination readiness (5), implemented a mass testing (“large-scale testing”) protocol and a proactive contact tracing campaign (29). The crisis communication has been overall very effective in Luxembourg (29). Due to having a clear crisis communication strategy in place before the pandemic, the crisis communication services were able to utilize numerous channels to reach a wide audience (29). They aimed to inform the population with clear public health messages disseminated, *inter alia*, directly to households via postal mail and in the five languages predominantly spoken in the country. Organizational strategies such as the Luxembourgish government sending invitations to eligible individuals might have also contributed to the high vaccine coverage, as this active intervention has been described as effective in the immunization campaign against Human papillomavirus in Italy (30). Convenient access to vaccination provided ample opportunities for vaccination, such as numerous vaccination centers spread across the country and a vaccination bus present at various events, like concerts or expositions (5, 30).

Recent findings hypothesized that a low level of vaccination willingness could be associated with a small number of COVID-19 cases and mortality reported in the media (31). At the time of the survey, in June 2021, Luxembourg experienced low rates of COVID-positive cases and mortality compared to other European countries (e.g., Italy and Portugal). This could be one further explanation why vaccination willingness in Luxembourg was not as high as that in other EU-countries according to our study, given that vaccination willingness has been shown increased with higher severity and proximity of personal experiences (32). Findings on determinants of vaccination hesitancy may vary in different cultural or socioeconomic contexts and may also depend on policies and the mode of administration of COVID-19 vaccines, such as mandatory vaccination certification for visiting public spaces like nightclubs (33) or administering COVID-19 boosters simultaneously with an influenza vaccine (34).

### 4.1 Limitations

To limit participant burden, only participants that were not yet vaccinated replied to the question on vaccination willingness. At the time of the survey, 67.0% of the respondents were already vaccinated. Therefore, we only analysed the results of those who were not yet vaccinated. The studies CON-VINCE and YAC did not collect data at the exact same point in time. For the CON-VINCE participants, the questionnaires were administered from April to June 2021 and, for the younger population, YAC, from August to September 2021. At the beginning of July 2021, an invitation for vaccination against SARS-CoV-2 was sent out to every adult resident of Luxembourg, eligible for vaccination (5). Children from 12 years and older received their invitation for vaccination from the 28th of June onwards (6). This slight timepoint difference however allowed minimization of the differences in eligibility to receive the vaccine. Furthermore, although the survey was conducted in mid-2021, it provides an important snapshot of beliefs toward the COVID-19 vaccination in the Luxembourgish population. Even though the sampling strategy strove for representativeness of the Luxembourgish resident population, we could not include the hard-to-reach non-resident population (e.g., individuals without internet access, homeless people, and undocumented migrants). Lastly, the sample size of some age groups, especially participants at older ages (aged over 60 years) that have not yet been vaccinated is quite small. Therefore, we are limited in the interpretation of reasons for vaccine hesitancy in this subgroup.

### 4.2 Outlook

An important question will be if COVID-19 vaccine hesitancy will provoke more general vaccination hesitancy in the future. Although trends have shown an increase of vaccination confidence in the safety and importance of vaccines in general since 2018 (35), we can expect that vaccine hesitancy may increase after the COVID-19 crisis if there is no clear rise in public awareness in the vaccines’ role to prevent severe disease, in contrast to widespread public beliefs that vaccines prevent infection. The context of the COVID-19 pandemic presents a complex context for vaccine confidence is concerned. Appearance of new variants mirrors the unpredictability of the situation, raising questions about the effectiveness of vaccination. Future research work should concentrate on this very important topic. Furthermore, future studies should concentrate on how we can proceed to avoid vaccine-fatigue and on the impact the booster vaccinations will have on vaccine fatigue.

Given that the European populations were recognized as being among the least vaccine confident in the world in 2016 (36), and the very diverse COVID-19 vaccination willingness in the European community, it is essential to continue to study vaccination willingness and to compare the reasons for and against COVID-19 vaccination on a wider level.

## 5 Conclusion

Using a large cross-sectional, population-representative sample of residents with a large age range across adolescence and adulthood in Luxembourg, we explored motivations for and against SARS-CoV-2 vaccination and defined population groups most at risk for COVID-19 vaccination hesitancy.

Our findings highlight that vaccine hesitancy represents an important challenge, and reasons for vaccination hesitancy differ according to the age of the respondents. Our results help improve future pandemic preparedness by identifying socio-demographic groups most likely to be hesitant or reluctant towards vaccination and thus providing guidance for the adaptation of public health messaging to reach these groups. Through this, we contribute to the improvement of strategies aiming at increasing vaccination uptake.

## Data Availability

The dataset for this manuscript is not publicly available as it contains sensitive data. Any reasonable requests for accessing the CON-VINCE dataset can be directed to con-vince@lih.lu. Data of the YAC study can be accessed upon reasonable request to robin.samuel@uni.lu. All data of the manuscript will be provided upon approval by the ethics committee.

## CON-VINCE consortium and ORCHESTRA working group

Tamir Abdelrahman, Laboratoire National de Santé, Dudelange, Luxembourg; Geeta Acharya, Luxembourg Institute of Health, Strassen, Luxembourg; Gloria Aguayo, Luxembourg Institute of Health, Strassen, Luxembourg; Pinar Alper, Luxembourg Centre for Systems Biomedicine, University of Luxembourg, Esch-Belval, Luxembourg; Wim Ammerlaan, Luxembourg Institute of Health, Strassen, Luxembourg; François Ancien, Luxembourg Centre for Systems Biomedicine, University of Luxembourg, Esch-Belval, Luxembourg; Ariane Assele-Kama, Luxembourg Institute of Health, Strassen, Luxembourg; Christelle Bahlawane, Luxembourg Institute of Health, Strassen, Luxembourg; Katy Beaumont, Luxembourg Institute of Health, Strassen, Luxembourg; Nadia Beaupain, Luxembourg Institute of Health, Strassen, Luxembourg; Lucrèce Beckers, Luxembourg Institute of Health, Strassen, Luxembourg; Camille Bellora, Luxembourg Institute of Health, Strassen, Luxembourg; Fay Betsou, Luxembourg Institute of Health, Strassen, Luxembourg; Luc Biver, TNS-ILRES, Bertrange, Luxembourg; Sandie Boly, Luxembourg Institute of Health, Strassen, Luxembourg; Dirk Brenner, Luxembourg Institute of Health, Strassen, Luxembourg; Henry-Michel Cauchie, Luxembourg Institute of Science and Technology, Luxembourg, Luxembourg; Eleftheria Charalambous, Luxembourg Institute of Health, Strassen, Luxembourg; Emilie Charpentier, Luxembourg Institute of Health, Strassen, Luxembourg; Estelle Coibion, Laboratoire National de Santé, Dudelange, Luxembourg; Sylvie Coito, Ketterthill, Belvaux, Luxembourg; Delphine Collart, Luxembourg Institute of Science and Technology, Luxembourg, Luxembourg; Manuel Counson, Luxembourg Institute of Health, Strassen, Luxembourg; Brian De Witt, Luxembourg Institute of Health, Strassen, Luxembourg; Antonelle Di Pasquale, TNS-ILRES, Bertrange, Luxembourg; Olivia Domingues, Luxembourg Institute of Health, Strassen, Luxembourg; Claire Dording, Luxembourg Institute of Health, Strassen, Luxembourg; Jean-Luc Dourson, BioNeXt, Leudelange, Luxembourg; Bianca Dragomir, Luxembourg Institute of Health, Strassen, Luxembourg; Tessy Fautsch, Luxembourg Institute of Health, Strassen, Luxembourg; Jean-Yves Ferrand, Luxembourg Institute of Health, Strassen, Luxembourg; Thibault Ferrandon, BioNeXt, Leudelange, Luxembourg; Ana Festas Lopes, Luxembourg Institute of Health, Strassen, Luxembourg; Guillaume Fournier, Laboratoire National de Santé, Dudelange, Luxembourg; Joëlle Véronique Fritz, Luxembourg Institute of Health, Strassen, Luxembourg; Manon Gantenbein, Luxembourg Institute of Health, Strassen, Luxembourg; Piotr Gawron, Luxembourg Centre for Systems Biomedicine, University of Luxembourg, Esch-Belval, Luxembourg; Laura Georges, Luxembourg Institute of Health, Strassen, Luxembourg; Soumyabrata Ghosh, Luxembourg Centre for Systems Biomedicine, University of Luxembourg, Esch-Belval, Luxembourg; Stéphane Gidenne, Ketterthill, Belvaux, Luxembourg; Enrico Glaab, Luxembourg Centre for Systems Biomedicine, University of Luxembourg, Esch-Belval, Luxembourg; Clarissa P. C. Gomes, Luxembourg Centre for Systems Biomedicine, University of Luxembourg, Esch-Belval, Luxembourg; Borja Gomez Ramos, Luxembourg Centre for Systems Biomedicine, University of Luxembourg, Esch-Belval, Luxembourg; Vyron Gorgogietas, Luxembourg Centre for Systems Biomedicine, University of Luxembourg, Esch-Belval, Luxembourg; Jérôme Graas, Luxembourg Institute of Health, Strassen, Luxembourg; Valentin Groues, Luxembourg Centre for Systems Biomedicine, University of Luxembourg, Esch-Belval, Luxembourg; Wei Gu, Luxembourg Centre for Systems Biomedicine, University of Luxembourg, Esch-Belval, Luxembourg; Gael Hamot, Luxembourg Institute of Health, Strassen, Luxembourg; Anne-Marie Hanff, Luxembourg Institute of Health, Strassen, Luxembourg; Maxime Hansen, Luxembourg Institute of Health, Strassen, Luxembourg; Linda Hansen, Centre Hospitalier de Luxembourg, Luxembourg, Luxembourg; Lisa Hefele, Luxembourg Institute of Health, Strassen, Luxembourg; Laurent Heirendt, Luxembourg Centre for Systems Biomedicine, University of Luxembourg, Esch-Belval, Luxembourg; Ahmed Hemedan, Luxembourg Centre for Systems Biomedicine, University of Luxembourg, Esch-Belval, Luxembourg; Estelle Henry, Luxembourg Institute of Health, Strassen, Luxembourg; Margaux Henry, Luxembourg Institute of Health, Strassen, Luxembourg; Eve Herkenne, Luxembourg Institute of Health, Strassen, Luxembourg; Sascha Herzinger, Luxembourg Centre for Systems Biomedicine, University of Luxembourg, Esch-Belval, Luxembourg; Christiane Hilger, Luxembourg Institute of Health, Strassen, Luxembourg; Laetitia Huiart, Luxembourg Institute of Health, Strassen, Luxembourg; Alexander Hundt, Luxembourg Institute of Health, Strassen, Luxembourg; Judith Hübschen, Luxembourg Institute of Health, Strassen, Luxembourg; Gilles Iserentant, Luxembourg Institute of Health, Strassen, Luxembourg; Philipp Jägi, Laboratoire Réunis, Junglinster, Luxembourg; Anne Kaysen, Luxembourg Centre for Systems Biomedicine, University of Luxembourg, Esch-Belval, Luxembourg; Piyapong Khurmin, Luxembourg Centre for Systems Biomedicine, University of Luxembourg, Esch-Belval, Luxembourg; Fédéric Klein, BioNeXt, Leudelange, Luxembourg; Tommy Klein, TNS-ILRES, Bertrange, Luxembourg; Stéphanie Kler, Luxembourg Institute of Health, Strassen, Luxembourg; Alexey Kolodkin, Luxembourg Institute of Health, Strassen, Luxembourg; Rejko Krüger, Luxembourg Institute of Health, Strassen, Luxembourg; Pauline Lambert, Luxembourg Institute of Health, Strassen, Luxembourg; Jacek Jaroslaw Lebioda, Luxembourg Centre for Systems Biomedicine, University of Luxembourg, Esch-Belval, Luxembourg; Sabine Lehmann, Luxembourg Institute of Health, Strassen, Luxembourg; Marie Leick, Laboratoire National de Santé, Dudelange, Luxembourg; Anja Leist, University of Luxembourg, Esch-sur-Alzette, Luxembourg; Morgane Lemaire, Luxembourg Institute of Health, Strassen, Luxembourg; Andrew Lumley, Luxembourg Institute of Health, Strassen, Luxembourg; Annika Lutz, University of Luxembourg, Esch-sur-Alzette, Luxembourg; João Manuel Loureiro, Centre Hospitalier de Luxembourg, Luxembourg, Luxembourg; Monica Marchese, Luxembourg Institute of Health, Strassen, Luxembourg; Tainà Marques, Luxembourg Centre for Systems Biomedicine, University of Luxembourg, Esch-Belval, Luxembourg; François Massart, Luxembourg Centre for Systems Biomedicine, University of Luxembourg, Esch-Belval, Luxembourg; Patrick May, Luxembourg Centre for Systems Biomedicine, University of Luxembourg, Esch-Belval, Luxembourg; Maura Minelli, Luxembourg Institute of Health, Strassen, Luxembourg; Alessandra Mousel, Luxembourg Institute of Health, Strassen, Luxembourg; Maeva Munsch, Luxembourg Institute of Health, Strassen, Luxembourg; Sophie Mériaux, Luxembourg Institute of Health, Strassen, Luxembourg; Friedrich Mühlschlegel, Laboratoire National de Santé, Dudelange, Luxembourg; Mareike Neumann, Luxembourg Institute of Health, Strassen, Luxembourg; Trang Nguyen, Luxembourg Institute of Health, Strassen, Luxembourg; Beatrice Nicolai, Centre Hospitalier de Luxembourg, Luxembourg, Luxembourg; Marc Paul O′Sullivan, Luxembourg Institute of Health, Strassen, Luxembourg; Leslie Ogorzaly, Luxembourg Institute of Science and Technology, Luxembourg, Luxembourg; Jochen Ohnmacht, Luxembourg Institute of Health, Strassen, Luxembourg; Christiane Olesky, Luxembourg Centre for Systems Biomedicine, University of Luxembourg, Esch-Belval, Luxembourg; Markus Ollert, Luxembourg Institute of Health, Strassen, Luxembourg; Claire Pauly, Luxembourg Centre for Systems Biomedicine, University of Luxembourg, Esch-Belval, Luxembourg; Laure Pauly, Luxembourg Centre for Systems Biomedicine, University of Luxembourg, Esch-Belval, Luxembourg; Lukas Pavelka, Luxembourg Centre for Systems Biomedicine, University of Luxembourg, Esch-Belval, Luxembourg; Christian Penny, Luxembourg Institute of Science and Technology, Luxembourg, Luxembourg; Magali Perquin, Luxembourg Institute of Health, Strassen, Luxembourg; Achilleas Pexaras, Luxembourg Institute of Health, Strassen, Luxembourg; Palma di Pinto, Ketterthill, Belvaux, Luxembourg; Marie France Pirard, Laboratoire National de Santé, Dudelange, Luxembourg; Jean-Marc Plesseria, Luxembourg Institute of Health, Strassen, Luxembourg; Guilherme Ramos Meyers, Luxembourg Centre for Systems Biomedicine, University of Luxembourg, Esch-Belval, Luxembourg; Armin Rauschenberger, Luxembourg Centre for Systems Biomedicine, University of Luxembourg, Esch-Belval, Luxembourg; Lucie Remark, Luxembourg Institute of Health, Strassen, Luxembourg; Antonio Rodriguez, TNS-ILRES, Bertrange, Luxembourg; Basile Rommes, Luxembourg Centre for Systems Biomedicine, University of Luxembourg, Esch-Belval, Luxembourg; Kirsten Rump, Luxembourg Centre for Systems Biomedicine, University of Luxembourg, Esch-Belval, Luxembourg; Estelle Sandt, Luxembourg Institute of Health, Strassen, Luxembourg; Bruno Santos, Luxembourg Institute of Health, Strassen, Luxembourg; Venkata P. Satagopam, Luxembourg Centre for Systems Biomedicine, University of Luxembourg, Esch-Belval, Luxembourg; Aurélie Sausy, Luxembourg Institute of Health, Strassen, Luxembourg; Margaux Schmitt, Luxembourg Institute of Health, Strassen, Luxembourg; Christiane Schmitt, Laboratoire Réunis, Junglinster, Luxembourg; Reinhard Schneider, Luxembourg Centre for Systems Biomedicine, University of Luxembourg, Esch-Belval, Luxembourg; Valerie Schröder, Luxembourg Centre for Systems Biomedicine, University of Luxembourg, Esch-Belval, Luxembourg; Serge Schumacher, TNS-ILRES, Bertrange, Luxembourg; Alexandra Schweicher, Centre Hospitalier de Luxembourg, Luxembourg, Luxembourg; Sneeha Seal, Luxembourg Institute of Health, Strassen, Luxembourg; Jean-Yves Servais, Luxembourg Institute of Health, Strassen, Luxembourg; Florian Simon, Luxembourg Institute of Health, Strassen, Luxembourg; Amna Skrozic, Luxembourg Centre for Systems Biomedicine, University of Luxembourg, Esch-Belval, Luxembourg; Chantal Snoeck, Luxembourg Institute of Health, Strassen, Luxembourg; Kate Sokolowska, Luxembourg Institute of Health, Strassen, Luxembourg; Lara Stute, Luxembourg Centre for Systems Biomedicine, University of Luxembourg, Esch-Belval, Luxembourg; Hermann Thien, Luxembourg Institute of Health, Strassen, Luxembourg; Stéphane Toll, Laboratoire Réunis, Junglinster, Luxembourg; Noua Toukourou, Luxembourg Centre for Systems Biomedicine, University of Luxembourg, Esch-Belval, Luxembourg; Christophe Trefois, Luxembourg Centre for Systems Biomedicine, University of Luxembourg, Esch-Belval, Luxembourg; Johanna Trouet, Luxembourg Institute of Health, Strassen, Luxembourg; Nguyen Trung, Laboratoire National de Santé, Dudelange, Luxembourg; Jonathan Turner, Luxembourg Institute of Health, Strassen, Luxembourg; Michel Vaillant, Luxembourg Institute of Health, Strassen, Luxembourg; Daniela Valoura Esteves, Luxembourg Institute of Health, Strassen, Luxembourg; Carlos Vega Moreno, Luxembourg Centre for Systems Biomedicine, University of Luxembourg, Esch-Belval, Luxembourg; Charlène Verschueren, Luxembourg Institute of Health, Strassen, Luxembourg; Maharshi Vyas, Luxembourg Centre for Systems Biomedicine, University of Luxembourg, Esch-Belval, Luxembourg; Claus Vögele, University of Luxembourg, Esch-sur-Alzette, Luxembourg; Cécile Walczak, Luxembourg Institute of Science and Technology, Luxembourg, Luxembourg; Xinhui Wang, Luxembourg Centre for Systems Biomedicine, University of Luxembourg, Esch-Belval, Luxembourg; Femke Wauters, Centre Hospitalier de Luxembourg, Luxembourg, Luxembourg; Bernard Weber, Laboratoire Réunis, Junglinster, Luxembourg; Emilie Weibel, BioNeXt, Leudelange, Luxembourg; Tania Zamboni, Luxembourg Institute of Health, Strassen, Luxembourg.

## Author disclaimer

The views expressed in this paper are the sole responsibility of the authors and the Commission is not responsible for any use that may be made of the information it contains. For the purpose of open access, and in fulfilment of the obligations arising from the grant agreement, the author has applied a Creative Commons Attribution 4.0 International (CC BY 4.0) license to any Author Accepted Manuscript version arising from this submission.
