# Supplementary material for: Lessons learned from the COVID-19 pandemic: identifying hesitant groups and exploring reasons for vaccination hesitancy, from adolescence to late adulthood
Source: Front Public Health. 2024 Dec 24;12:1456265. doi: 10.3389/fpubh.2024.1456265 (PMC11705566; doi:10.3389/fpubh.2024.1456265)
Supplement: Supplementary file 1 [file Data_Sheet_1.pdf]

Supplementary Material

Lessons learned from the COVID-19 pandemic: identifying hesitant groups and exploring reasons for vaccination hesitancy, from adolescence to late adulthood

Laure Pauly 1,2,3,4, Caroline Residori 5, Hamid Bulut 5, Dmitry Bulaev 6, Soumyabrata Ghosh 3, Marc P. O'Sullivan 1, Joëlle V. Fritz 1, Michel Vaillant 6, Basile Rommes 3, Robin Samuel 5, Venkata P. Satagopam 3, Rejko Krüger 1,3,4* and Anja K. Leist 5* on behalf of the CON-VINCE Consortium and the ORCHESTRA Working Group

1 Transversal Translational Medicine, Luxembourg Institute of Health, Strassen, Luxembourg, 2 Faculty of Science, Technology and Medicine, University of Luxembourg, Esch-sur-Alzette, Luxembourg, 3 Luxembourg Centre for Systems Biomedicine, University of Luxembourg, Esch-sur-Alzette, Luxembourg, 4 Parkinson Research Clinic, Centre Hospitalier de Luxembourg, Strassen, Luxembourg, 5 Department of Social Sciences, University of Luxembourg, Esch-sur-Alzette, Luxembourg, 6 Competence Centre for Methodology and Statistics, Luxembourg Institute of Health, Strassen, Luxembourg

***Correspondence:**

Anja K. Leist: anja.leist@uni.lu

Rejko Krüger: rejko.krüger@lih.lu

1 Supplementary Data

A.0. Baseline demographic characteristics by cohort

Characteristic	YAC<br>No. 3,740	CON-VINCE<br>No. 1,865	p-value
**Gender, n (%)**			**0.26**
Female	1960 (52%)	946 (51%)	
Male	1780 (48%)	917 (49%)	
Missing	0 (0%)	2 (0%)	
**Age, median (IQR)**	20 (16 - 25)	47 (36 - 59)	**< 0.0001**
**Origin, n (%)**			**< 0.0001**
Luxembourg	2245 (60%)	1437 (77%)	
Belgium / Germany / Netherlands / France	367 (10%)	274 (15%)	
Italy / Portugal / Spain	320 (9%)	102 (5%)	
Other	516 (14%)	52 (3%)	
Missing	292 (8%)	0 (0%)	
**Marital status, n (%)**			**< 0.0001**
Single	2068 (55%)	325 (17%)	
Married	160 (4%)	895 (48%)	

Registered partnership	164 (4%)	156 (8%)	
Divorced	25 (1%)	129 (7%)	
Widowed	0 (0%)	47 (3%)	
Other	0 (0%)	26 (1%)	
Missing	1323 (35%)	287 (15%)	
**Children, n (%)**			**< 0.0001**
0	2341 (63%)	0 (0%)	
1	88 (2%)	277 (15%)	
2	26 (1%)	554 (30%)	
3	2 (0%)	207 (11%)	
4	0 (0%)	35 (2%)	
5	0 (0%)	17 (1%)	
6	1 (0%)	4 (0%)	
Missing	1282 (34%)	771 (41%)	
**Education degree, n (%)**			**< 0.0001**
No formal degree	42 (1%)	22 (1%)	
Primary or lower secondary education	715 (19%)	29 (2%)	

Secondary education – Classical system	535 (14%)	275 (15%)	
Secondary education – Technical system	640 (17%)	405 (22%)	
University degree	411 (11%)	730 (39%)	
Masters or Doctorate degree	424 (11%)	0 (0%)	
Other type of degree	14 (0%)	117 (6%)	
Missing	959 (26%)	287 (15%)	
**Employment status, n (%)**			**< 0.0001**
Full-time employed	902 (24%)	706 (38%)	
Part-time employed	80 (2%)	197 (11%)	
Unemployed	185 (5%)	13 (1%)	
In training / retraining / education	2377 (64%)	42 (2%)	
Parental leave	54 (1%)	82 (4%)	
Retirement or early retirement	0 (0%)	416 (22%)	
Looking after home or family	19 (1%)	15 (1%)	
Other	100 (3%)	107 (6%)	
Missing	23 (1%)	287 (15%)	
**Number of individuals in household, n (%)**			**< 0.0001**

1	383 (10%)	217 (12%)	
2	600 (16%)	576 (31%)	
3	704 (19%)	309 (17%)	
4	1067 (29%)	338 (18%)	
5 or more	725 (19%)	138 (7%)	
Missing	261 (7%)	287 (15%)	
**Height, median (IQR)**	170 (163 - 178)	172 (166 - 180)	**< 0.0001**
Missing	557 (15%)	0 (0%)	
**Weight, median (IQR)**	65 (55 - 75)	78 (67 - 90)	**< 0.0001**
Missing	422 (11%)	0 (0%)	
**SARS-CoV-2 tested positive**			**< 0.0001**
No	2541 (68%)	1408 (75%)	
Yes	581 (16%)	170 (9%)	
Missing	618 (17%)	287 (15%)	
**Household member SARS-CoV-2 tested positive**			**< 0.0001**
No	2350 (63%)	63 (3%)	
Yes	756 (20%)	106 (6%)	
Missing	634 (17%)	1696 (91%)	

Table A.0. Baseline demographic characteristic of the cohorts. For categorical variables, the p-values from Fisher's exact test are reported; for continuous variables the p-values from Wilcoxon-Mann-Whitney test are provided. *Source: YAC 2021 (n = 3740); CON-VINCE April 2020 (n = 1865).*

A.1. Summary statistics and regression results for vaccination status

Characteristic	N	Vaccinated, n (%)	OR	SE (OR)	OR 95% CI	p-value
Age Group: 12-15 - Reference Group	710	420 (59.15)				
Age Group: 16-19	718	543 (75.63)	2.29	0.27	[1.82, 2.88]	<0.001 ***
Age Group: 20-24	893	668 (74.80)	2.99	0.38	[2.34, 3.82]	<0.001 ***
Age Group: 25-29	1041	759 (72.91)	3.71	0.60	[2.70, 5.10]	<0.001 ***
Age Group: 30-39	289	88 (30.45)	7.23	1.99	[4.22, 12.38]	<0.001 ***
Age Group: 40-49	340	99 (29.12)	6.86	1.87	[4.02, 11.70]	<0.001 ***
Age Group: 50-59	326	205 (62.88)	25.34	6.94	[14.81, 43.34]	<0.001 ***
Age Group: 60+	419	394 (94.03)	102.94	39.65	[48.38, 219.01]	<0.001 ***
Gender: Female - Reference Group	2470	1649 (66.76)				
Gender: Male	2266	1527 (67.39)	1.03	0.07	[0.90, 1.19]	0.659
Employment status: Full-time employed - Reference Group	1475	867 (58.78)				
Employment status: Part-time employed	258	136 (52.71)	1.07	0.17	[0.78, 1.46]	0.686
Employment status: Unemployed	173	108 (62.43)	0.49	0.09	[0.34, 0.71]	<0.001 ***
Employment status: In vocational training / education	2075	1480 (71.33)	1.11	0.16	[0.84, 1.47]	0.472
Employment status: Parental leave / Looking after family	126	82 (65.08)	1.14	0.26	[0.73, 1.79]	0.560
Employment status: In retirement / early retirement	416	390 (93.75)	3.26	0.99	[1.80, 5.93]	<0.001 ***
Employment status: Chronic illness or disability	28	16 (57.14)	0.65	0.28	[0.28, 1.49]	0.307
Employment status: Other	185	97 (52.43)	0.80	0.15	[0.56, 1.16]	0.240
Origin: LU – Reference Group	3293	2251 (68.36)				
Origin: DE / NL / FR / BE	574	378 (65.85)	0.75	0.08	[0.60, 0.93]	0.008 **
Origin: IT / SP / PO	368	226 (61.41)	0.63	0.08	[0.49, 0.80]	<0.001 ***
Origin: Other	501	321 (64.07)	0.57	0.06	[0.46, 0.71]	<0.001 ***

Table A.1. Descriptive statistics for all levels of fixed effects variables are provided as frequencies and proportions. The results of Generalised Linear Mixed effects Model for vaccination status are given as ORs, with corresponding CIs and p-values. *Source: YAC 2021 (n = 3160); CON-VINCE April – June 2021 (n = 1576). Abbreviations: OR = Odds Ratio; CI = Confidence Interval; LU = Luxembourg; DE = Germany; NL = Netherlands; FR = France; BE = Belgium; IT = Italy; SP = Spain; PO = Portugal.* *** p-value<0.001, ** p-value<0.01, * p-value<0.05

A.2. Vaccination scale-up against COVID-19, Luxembourg

Vaccination campaign	Age (years)	Date	Link
Phase 1	Nursing homes<br><br>Health prof.	06-01-2021	https://msan.gouvernement.lu/en/actualites.gouvernement%2Ben%2Bactualites%2Btoutes_actualites%2Bcommuniques%2B2021%2B01-janvier%2B06-vaccinations-maisons-soins.html
Phase 2	75+	17-02-2021	https://msan.gouvernement.lu/en/actualites.gouvernement%2Ben%2Bactualites%2Btoutes_actualites%2Bcommuniques%2B2021%2B02-fevrier%2B17-lancement-phase2-vaccination.html
Phase 3	70-74	12-03-2021	https://msan.gouvernement.lu/en/actualites.gouvernement%2Ben%2Bactualites%2Btoutes_actualites%2Bcommuniques%2B2021%2B03-mars%2B12-phase-3.html
Phase 4	65-69	30-03-2021	https://msan.gouvernement.lu/en/actualites.gouvernement%2Ben%2Bactualites%2Btoutes_actualites%2Bcommuniques%2B2021%2B03-mars%2B30-vaccination-phase4.html
Phase 5	55-64	07-04-2021	https://msan.gouvernement.lu/en/actualites.gouvernement%2Ben%2Bactualites%2Btoutes_actualites%2Bcommuniques%2B2021%2B04-avril%2B07-lancement-phase5-vaccination.html
phase 6	16-54	27-04-2021	https://msan.gouvernement.lu/en/actualites.gouvernement%2Ben%2Bactualites%2Btoutes_actualites%2Bcommuniques%2B2021%2B04-avril%2B27-lancement-phase6-vaccination.html
Phase 7	12-17	28-06-2021	https://msan.gouvernement.lu/en/actualites.gouvernement%2Ben%2Bactualites%2Btoutes_actualites%2Bcommuniques%2B2021%2B06-juin%2B28-invitations-vaccination.html

Table A.2. Vaccination scale-up against COVID-19, Luxembourg

A.3. Vaccination intentions

Intention	Sample frequency, n	Sample proportion, %	Population prevalence*, %	Pop. prevalence* 95% CI, %
Willing	913	58.2	73.7	[70.8-76.5]
Unlikely	419	26.7	15.3	[13.2-17.6]
Undecided	237	15.1	11.0	[9.1-13.1]
**Total**	**1569**	**100.0**	**100.0**	

Table A.3. Sample frequencies, sample proportions and estimated population prevalence of the vaccination intentions.
*Population prevalence was estimated via weighting the sample through post-stratification.

A.4.1. Reasons for vaccination willingness

Intention	Sample frequency, n	Sample proportion, %	Population prevalence*, %	Pop. prevalence* 95% CI, %
Protect myself	631	69.3	70.5	[66.5-74.2]
Protect vulnerable	298	32.7	31.3	[27.5-35.4]
Help society	681	74.8	78.9	[75.2-82.1]
Government recommended	137	15	13.7	[11-16.9]
Physician recommended	68	7.5	8.8	[6.6-11.6]
Employer recommended	24	2.6	3.6	[2.2-5.8]
Travel safely	478	52.5	54.1	[49.9-58.3]
Other	229	25.1	12.6	[10.2-15.4]
**Total**	**911**	**100**	**100**	

Table A.4.1. Sample frequencies, sample proportions and estimated population prevalence of the reasons for willingness to vaccinate.
*Population prevalence was estimated via weighting the sample through post-stratification.

A.4.2. Reasons for vaccination undecidedness

Intention	Sample frequency, n	Sample proportion, %	Population prevalence*, %	Pop. prevalence* 95% CI, %
No belief in any vaccination	22	9.6	3.7	[2.4-5.9]
Also do not get vaccinated against other diseases	10	4.4	5.2	[2.3-11.5]
Bad experience with other vaccines	10	4.4	6.5	[2.9-14]
Not informed enough about vaccination in general	21	9.2	4.5	[2.4-8.1]
Not informed enough about COVID vaccination	84	36.7	25.8	[18.7-34.4]
Not feeling at risk	48	21	13.7	[8.9-20.7]
Prefer to wait until more people are vaccinated	69	30.1	29.3	[21.3-38.7]
Afraid of side effects	120	52.4	67.9	[58.9-75.7]
Sceptical about effectiveness	67	29.3	28.7	[20.9-38]
Sceptical about effectiveness against future variants	83	36.2	44.2	[34.7-54.1]
Vaccine not tested sufficiently	136	59.4	68.9	[59.9-76.7]
Other	38	17	15.8	[9.9-24.3]
**Total**	**229**	**100**	**100**	

Table A.4.2. Sample frequencies, sample proportions and estimated population prevalence of the reasons for undecidedness to vaccinate.
*Population prevalence was estimated via weighting the sample through post-stratification.

A.4.3. Reasons for vaccination unlikeliness

Intention	Sample frequency, n	Sample proportion, %	Population prevalence*, %	Pop. prevalence* 95% CI, %
No belief in any vaccination	55	13.2	14.9	[10.1-21.3]
Also do not get vaccinated against other diseases	35	8.4	13	[7.9-20.7]
Bad experience with other vaccines	23	5.5	5.6	[3.1-9.9]
Not informed enough about vaccination in general	39	9.4	5.6	[3.8-8.3]
Not informed enough about COVID vaccination	136	32.6	32.4	[25.6-40.1]
Not feeling at risk	139	33.3	28.3	[22.2-35.2]
Prefer to wait until more people are vaccinated	87	20.9	19.6	[14.4-26]
Afraid of side effects	190	45.6	48.6	[41.1-56.2]
Sceptical about effectiveness	190	45.6	41.9	[34.7-49.5]
Sceptical about effectiveness against future variants	135	32.4	33.7	[27-41.1]
Vaccine not tested sufficiently	237	56.8	62.1	[54.7-69.0]
Other	62	15	15.7	[11.1-21.7]
**Total**	**413**	**100**	**100**	

Table A.4.3. Sample frequencies, sample proportions and estimated population prevalence of the reasons for unlikeliness to vaccinate.
*Population prevalence was estimated via weighting the sample through post-stratification.
